# Mechanism through Which Retrocyclin Targets Flavivirus Multiplication

**DOI:** 10.1128/JVI.00560-21

**Published:** 2021-07-12

**Authors:** Xiaoying Jia, Jiao Guo, Weirong Yuan, Lingling Sun, Yang Liu, Minmin Zhou, Gengfu Xiao, Wuyuan Lu, Alfredo Garzino-Demo, Wei Wang

**Affiliations:** a State Key Laboratory of Virology, Wuhan Institute of Virology, Center for Biosafety Mega-Science, Chinese Academy of Sciences, Wuhan, China; b University of the Chinese Academy of Sciences, Beijing, China; c Department of Microbiology and Immunology, the Institute of Human Virology, University of Maryland School of Medicine, Baltimore, Maryland, USA; d Department of Molecular Medicine, University of Padua, Padua, Italy; University of North Carolina at Chapel Hill

**Keywords:** retrocyclin-101, flavivirus, antiviral effect, NS2B-NS3 protease, DE loop

## Abstract

Currently, there are no approved drugs for the treatment of flavivirus infection. Accordingly, we tested the inhibitory effects of the novel θ-defensin retrocyclin-101 (RC-101) against flavivirus infection and investigated the mechanism underlying the potential inhibitory effects. First, RC-101 robustly inhibited both Japanese encephalitis virus (JEV) and Zika virus (ZIKV) infections. RC-101 exerted inhibitory effects on the entry and replication stages. Results also indicated that the nonstructural protein NS2B-NS3 serine protease might serve as a potential viral target. Furthermore, RC-101 inhibited protease activity at the micromolar level. We also demonstrated that with respect to the glycoprotein E protein of flavivirus, the DE loop of domain III (DIII), which is the receptor-binding domain of the E protein, might serve as another viral target of RC-101. Moreover, a JEV DE mutant exhibited resistance to RC-101, which was associated with deceased binding affinity of RC-101 to DIII. These findings provide a basis for the development of RC-101 as a potential candidate for the treatment of flavivirus infection.

**IMPORTANCE** Retrocyclin is an artificially humanized circular θ-defensin peptide, containing 18 residues, previously reported to possess broad antimicrobial activity. In this study, we found that retrocyclin-101 inhibited flavivirus (ZIKV and JEV) infections. Retrocyclin-101 inhibited NS2B-NS3 serine protease activity, suggesting that the catalytic triad of the protease is the target. Moreover, retrocyclin-101 bound to the DE loop of the E protein of flavivirus, which prevented its entry.

## INTRODUCTION

Flaviviruses are taxonomically classified in the genus *Flavivirus* and family *Flaviviridae*. These viruses include more than 70 different pathogens and are transmitted mostly by arthropods. Emerging and reemerging flaviviruses, such as Zika virus (ZIKV), Japanese encephalitis virus (JEV), dengue virus (DENV), West Nile virus (WNV), and yellow fever virus, cause public health problems worldwide (1). Flaviviruses contain an approximately 11-kb positive-stranded RNA genome that encodes three structural proteins, including the capsid (C), membrane (premembrane [prM] and membrane [M]), and envelope (E), as well as seven nonstructural proteins (NS1, NS2A, NS2B, NS3, NS4A, NS4B, and NS5) (2). The envelope glycoprotein (E) is responsible for receptor binding and membrane fusion and thus plays essential roles in virus entry. E proteins exist as homodimers on the surface of the virus. Among the three domains of the E protein, domain I (DI) connects the DII and DIII domains, and DII contains fusion polypeptides that facilitate membrane fusion, whereas DIII has been proposed to act as the receptor binding region (3–5). It has been reported that several key residues, such as the glycosylation site N154 and the DE loop (T_363_SSAN_367_), are responsible for receptor binding (6, 7), whereas H144 and H319 are thought to play critical roles in DI and DIII interactions (8). Moreover, Q258, located in DII, and T410, located in the stem, are indispensable for low-pH-triggered conformational changes, in which the stem region undergoes zippering along with DII, thus leading to the postfusion conformation and membrane fusion (9–11). As it envelops the surface of the virion, the E protein is the natural target for antibodies and the design of entry inhibitors to prevent receptor binding and membrane fusion (4, 9, 12, 13). Likewise, viral proteases such as NS2B-NS3 protease-helicase and the NS5 RNA-dependent RNA polymerase represent attractive drug targets in an attempt to identify replication inhibitors (14, 15).

**FIG 1:**
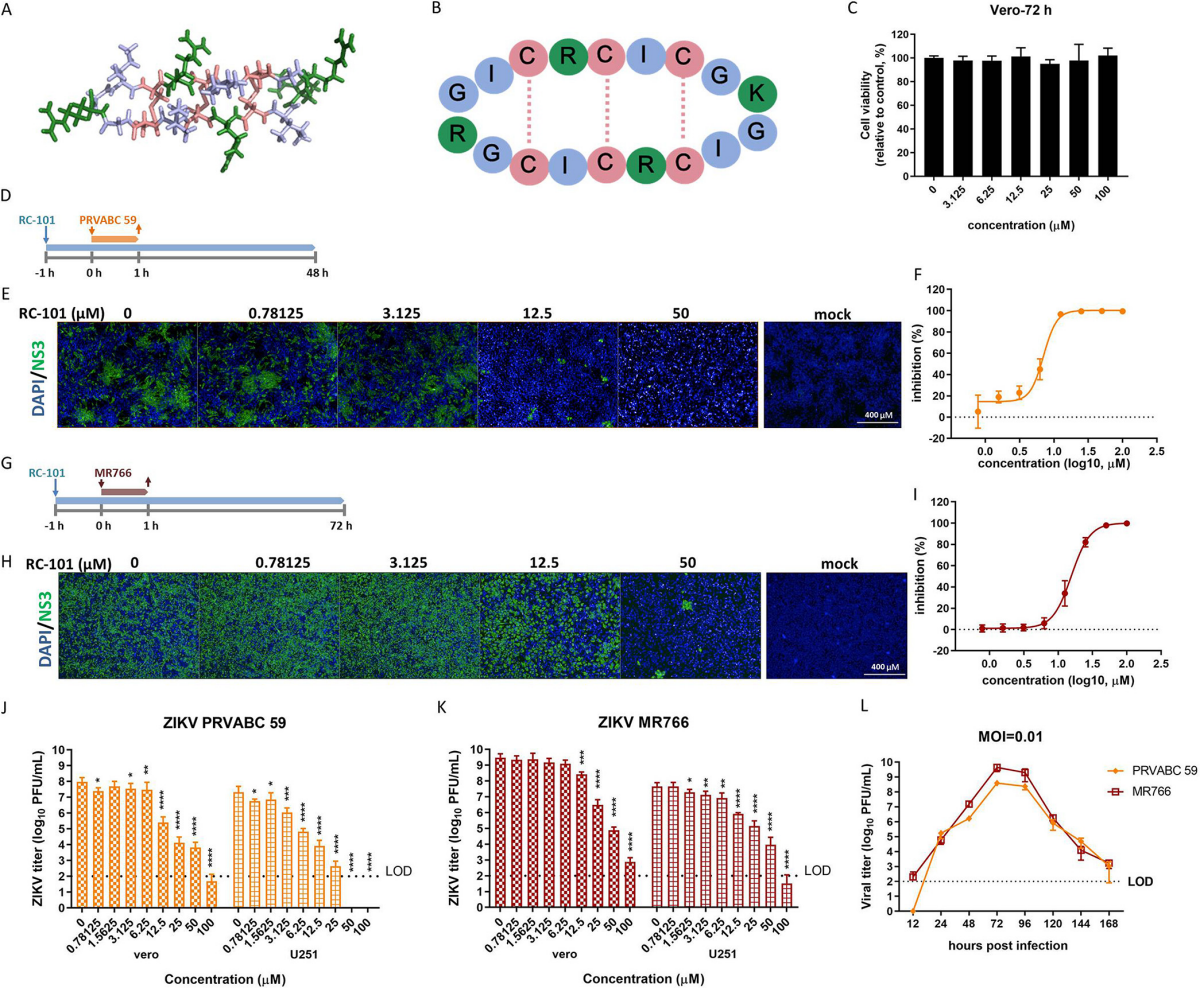
RC-101 inhibits ZIKV infection. (A) Stick diagram of the crystal structure of RC-2 (PDB no 2LZI). (B) Schematic diagram of RC-101. Colors in the schematic diagram correlate with those in panel A. (C) Cytotoxicity of RC-101. Vero cells were incubated with RC-101 at the indicated concentrations for 72 h. Cell viability was evaluated using the CCK-8 assay. (D) Timeline of IFA and plaque assays for PRVABC 59. Cells were incubated with RC-101 for 1 h at the indicated concentrations. ZIKV PRVABC 59 was then added at an MOI of 0.1 for 1 h. The cells were fixed and subjected to IFA assay, while the supernatant was subjected to plaque assay 47 h postinfection. (E) IFA images showing the ZIKV PRVABC 59 NS3 protein (green) and nuclei (blue) for Vero cells. (F) Dose-response curve of RC-101 for inhibition of ZIKV PRVABC 59 infection. (G) Timeline of IFA and plaque assays for ZIKV MR766. The procedure is the same as that in panel D, except ZIKV MR766 replaced PRVABC 59 and the supernatant was subjected to plaque assay for 71 h postinfection. (H) IFA images showing the ZIKV MR766 NS3 protein (green) and nuclei (blue) for Vero cells. (I) Dose-response curve of RC-101 for inhibition of ZIKV MR766 infection. (J) The inhibition of PRVABC 59 by RC-101 was determined using the plaque assay. (K) The inhibition of MR766 by RC-101 was determined using the plaque assay. (L) Growth kinetics of PRVABC 59 and MR766. Vero cells were infected at an MOI of 0.01 for 1 h. Supernatants were collected at the indicated time points postinfection and assayed for viral titer. Data are presented as the mean ± (standard deviation) SD from 3 to 8 independent experiments. LOD, limit of detection. *, *P* < 0.05; **, *P* < 0.01; ***, *P* < 0.001; ****, *P* < 0.0001.

Retrocyclin (RC) is an artificially humanized θ-defensin that has been reported to possess broad antimicrobial activity (16–21). RC-101 has the sequence GICRCICGKGICRCICGR and is an analogue of RC-1 (GICRCICGRGICRCICGR). It contains 18 residues, including three disulfide bonds and four positively charged residues (Fig. 1A and B), which confers high binding affinity to glycosylated proteins, such as HIV gp120 (22), influenza virus hemagglutinin (23), and herpes simplex virus 1/2 (HSV-1/2) glycoprotein (24), thus preventing virus entry. Additionally, some viral proteases with negatively charged surfaces might serve as targets for RC-1 (20).

In this study, we tested the inhibitory effect of RC-101 against flavivirus infection. As flaviviruses possess only one conserved N-linked glycan on the E protein (25), whether RC-101 exerted the inhibitory effect against flavivirus entry by targeting the glycan chain was tested in this study. Meanwhile, we determined that RC-101 could also inhibit flavivirus replication by blocking the NS2B-NS3 serine protease.

## RESULTS

### RC-101 inhibits ZIKV infection.

To test the inhibitory effect of RC-101 against ZIKV infection, two strains were used to determine the 50% inhibitory concentration (IC_50_) of RC-101. Notably, the ZIKV PRVABC 59 strain, belonging to the Asian lineage ZIKV strains, contains one N-linked glycosylation site (N-X-S/T) at residue N154 of E, which is conserved among the flaviviruses, whereas the stocks of the African lineage MR766 may or may not lack the E glycosylation motif due to their extensive passaging (26–31). To this end, an MR766 strain lacking the N-glycosylation motif (GenBank accession no. MK105975.1) was used in this study. The cytotoxicity of RC-101 was initially tested on Vero cells, which showed a marginal response even at 100 μM (Fig. 1C). An immunofluorescence antibody (IFA) staining plaque assay for the antiviral effect of RC-101 against ZIKV PRVABC 59 showed a dose-dependent inhibition, with an IC_50_ of 7.033 μM (Fig. 1D to F). Similarly, RC-101 inhibited ZIKV MR766 infection, with an IC_50_ of 15.58 μM (Fig. 1G to I). To verify the result, an additional cell line, the U251 glioma cell line, was used in the plaque assay. As shown in Fig. 1J, RC-101 robustly inhibited PRVABC 59 virus production; few plaques were found when 100 μM peptide was included, and an approximately 4- to 5-log unit reduction was found in the 12.5 μM treatment group. Similarly, RC-101 robustly inhibited MR766 virus production, with a reduction of approximately 7 log units when 100 μM peptide was used and a reduction of approximately 1 log unit when 12.5 μM RC-101 was used (Fig. 1K). To validate the comparison results, the replication kinetics of both strains were evaluated. As shown in Fig. 1L, both strains had similar growth curves, with an accumulation of infectious virions that reached the highest titer at 72 h postinfection.

### RC-101 inhibits ZIKV infection at both the entry and replication steps.

**FIG 2:**
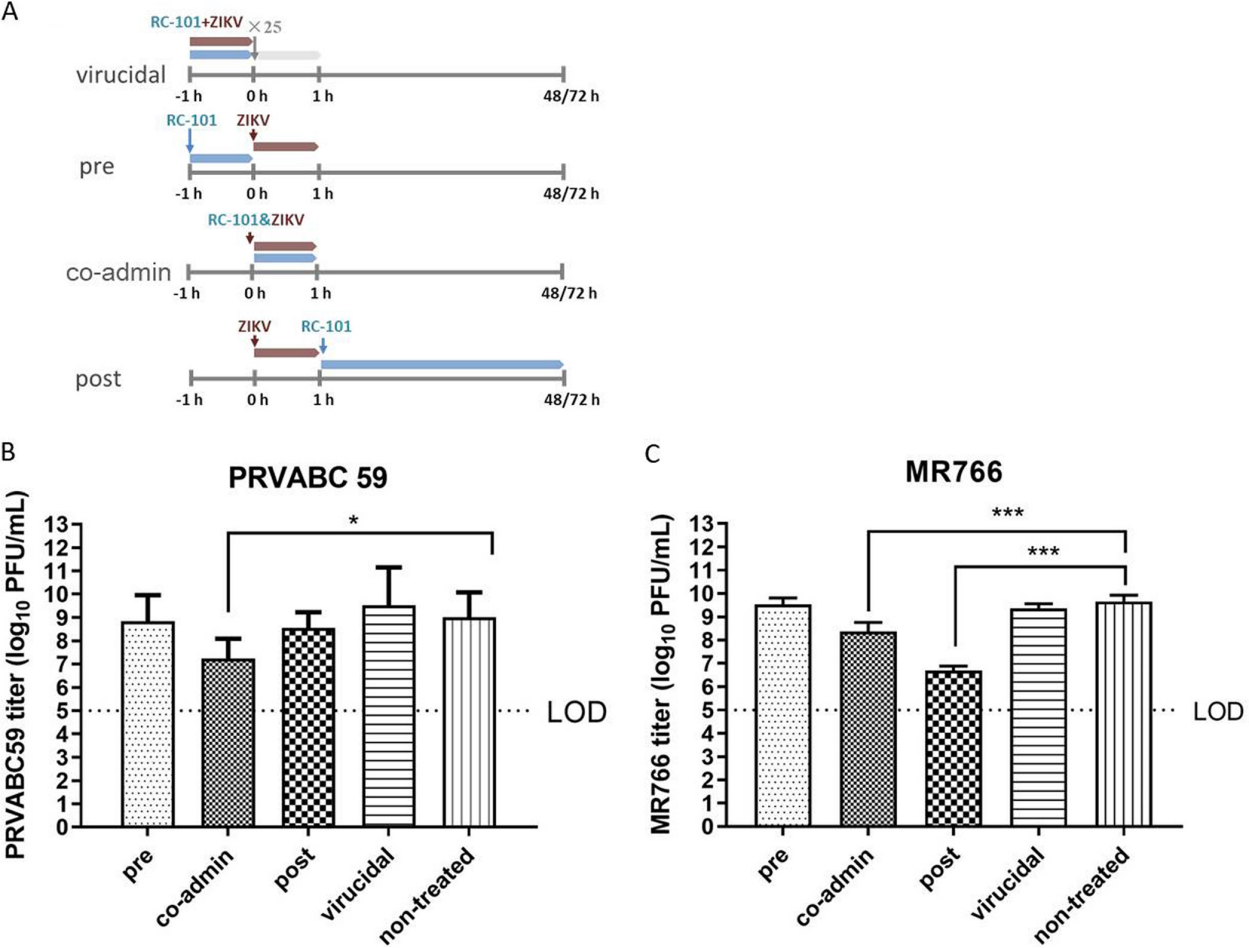
Time-of-addition analysis of the antiviral activity of the RC-101. (A) Schematic illustration of the time-of-addition experiment. For virucidal treatment, ZIKV (MOI of 2.5) was incubated with RC-101 (40 μM) at 37°C for 1 h, and the mixture was diluted 25-fold to infect Vero cells for 1 h. For pretreatment (pre), Vero cells were incubated with RC-101 (40 μM) for 1 h (from −1 to 0 h) and then infected with ZIKV (MOI of 0.1) for 1 h (from 0 to 1 h). co-admin, coadministration treatment. Vero cells were incubated with a mixture of RC-101 (40 μM) and ZIKV (MOI of 0.1) for 1 h (0 to 1 h). Posttreatment, Vero cells were infected with ZIKV (MOI of 0.1) for 1 h and then incubated with RC-101 (40 μM) for an additional 47 h (PRVABC 59) and 71 h (MR766), respectively. (B and C) Time-of-addition analysis of the antiviral effect of RC-101 against PRVABC 59 (B) and MR766 (C) The inhibitory effect of the drugs in each group was determined by plaque assays. Data are presented as the mean ± SD from 5 to 8 independent experiments. LOD, limit of detection. *, *P* < 0.05; ***, *P* < 0.001.

To test whether RC-101 blocked the entry step or the replication step, a time-of-addition experiment was performed (Fig. 2A). As shown in Fig. 2B and C, no suppression of viral titers was observed in the pretreatment or the virucidal treatment groups, indicating that RC-101 does not inhibit ZIKV infection—either by blocking the cellular receptors that prevent virus binding or by inactivating the virus directly. However, RC-101 exerted significant inhibitory effects when its addition was synchronized with the virus via coadministration. Moreover, RC-101 inhibited MR766 strain infection when it was added 1 h postinfection. These results suggested that viral entry and replication are the stages at which RC-101 shows inhibitory activity.

**FIG 3:**
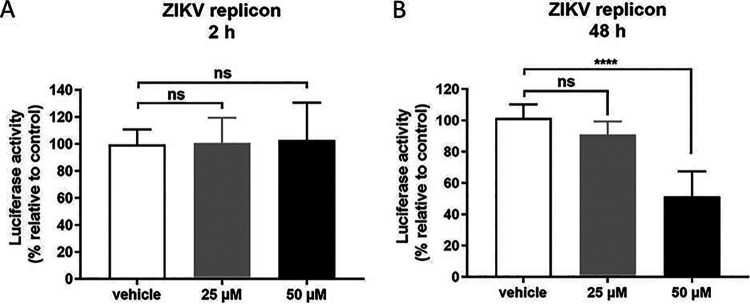
RC-101 inhibits Zika virus (ZIKV) replicon activity. (A and B) BHK-21 cells transfected with the ZIKV replicon were treated with RC-101, and luciferase activities were determined at 2 h (B) and 48 h (C). Data are presented as the mean ± SD from three independent experiments. ns, not significant; ****, *P* < 0.0001.

To confirm the inhibitory effect on viral replication, we investigated the effects of RC-101 on the ZIKV replicon (32, 33). As shown in Fig. 3, RC-101 showed little effect on the initial translation of replicon RNA (Fig. 3A), whereas an appreciable reduction in the luciferase signal was observed at 48 h postelectroporation (Fig. 3B). This confirmed that RC-101 has an inhibitory effect on the ZIKV replication state.

### RC-101 inhibits NS2B-NS3 serine protease activity.

**FIG 4:**
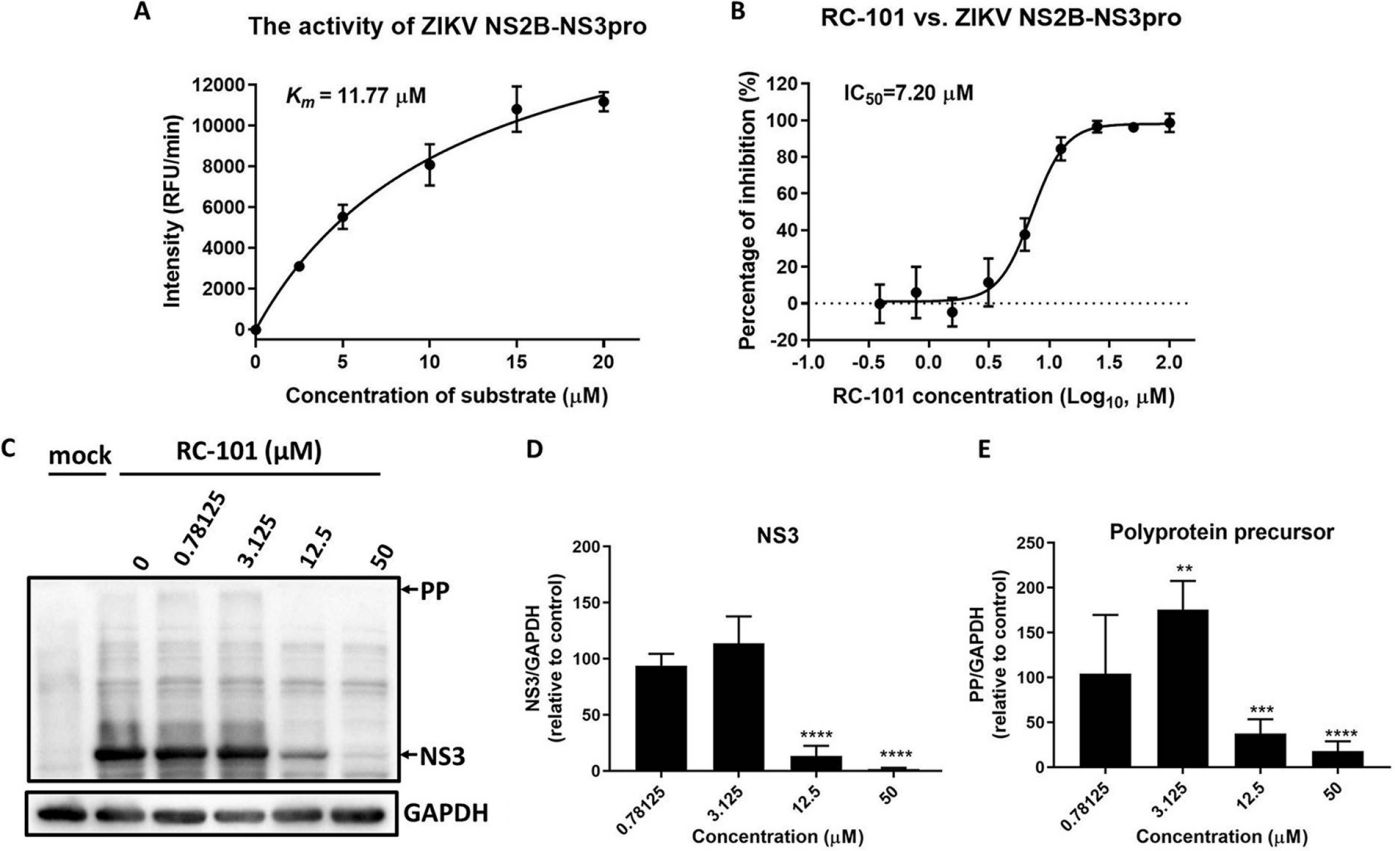
RC-101 inhibits NS2B-NS3 serine protease activity. (A) Enzyme kinetic assay of NS2B-NS3pro activity. The fluorogenic substrate peptide Boc-Gly-Arg-Arg-AMC was serially diluted to assess the activity of Zika virus (ZIKV) protease. The relative fluorescence units (RFU) were measured using an EnSpire multimode plate reader with emission at 440 nm upon excitation at 350 nm. (B) Inhibitory effect of RC-101 against the activity of ZIKV NS2B-NS3pro. The reaction mixtures of NS2B-NS3pro (100 μl) consisted of 12 μM substrate peptide, 1.25 μM NS2B-NS3pro, and RC-101 at various concentrations with a buffer comprised of 200 mM Tris-HCl (pH 8.5), and this was incubated at 37°C for 30 min. (C) Western blot analysis of the inhibition of JEV NS3 protease activity by RC-101. BHK-21 cells were incubated with RC-101 at the indicated concentrations, with a 1-h preinfection, before infection with JEV AT31 at an MOI of 0.1 for 1 h. The cell lysates were subjected to Western blotting 23 h postinfection. (D) NS3 expression relative to the control. (E) Polyprotein precursor expression relative to the control. Data are presented as the mean ± SD from 4 to 6 independent experiments. **, *P* < 0.01; ***, *P* < 0.001; ****, *P* < 0.0001.

To investigate the potential viral target of RC-101, we tested the inhibitory effect of RC-101 on ZIKV NS2B-NS3 protease activity. It has been reported that RC-1, which possesses the same residue sequence as RC-101, except for one lysine (K) instead of arginine (R) in RC-101, might dock at the NS2B-NS3 interface and thus inhibit DENV-2 replication by interfering with the activity of the NS2B-NS3 serine protease (20). Considering the sequence and structural conservation of flavivirus NS proteins, we reasoned that RC-101 might have a similar effect on the ZIKV NS2B-NS3 protease. To test this hypothesis, we first produced NS2B-NS3pro in Escherichia coli as a single-chain peptide (20, 34, 35). Protease activity was assessed using a fluorogenic peptide as a substrate at 37°C for 30 min. As shown in Fig. 4A, the Michaelis-Menten constant (*K_m_*) value was 11.77 μM, indicating that the enzyme kinetic assay was robust and suitable to investigate the inhibitory effect. As shown in Fig. 4B, RC-101 effectively inhibited NS2B-NS3 protease activity, with an IC_50_ of 7.20 μM, indicating that this protease serves as a viral target of RC-101.

Inhibition of the protease activity of NS3 by RC-101 was further supported by the detection of the unprocessed polyprotein precursor (PP) and NS3 in the infected cells (36). As shown in Fig. 4C to D, the expression of JEV NS3 (∼70 kDa) was inhibited in a dose-dependent manner by RC-101. Notably, the unprocessed polyprotein precursor (>180 kDa) was present in the low-RC-101-concentration groups (0.78125 and 3.125 μM), and the level of the polyprotein precursor at 3.125 μM was significantly higher than that at 0.78125 μM, indicating that the protease activity of NS3 was inhibited at these RC-101 concentrations. The presence of the polyprotein precursor decreased in the high-RC-101-concentration groups (12.5 and 50 μM), since the viral infection was robustly blocked in these groups (Fig. 4C and E). Based on both the *in vitro* enzyme kinetic assays and the experiments in infected cells, it was concluded that RC-101 inhibits flavivirus NS2B-NS3 serine protease activity.

### RC-101 inhibits flavivirus entry by targeting the DE loop of E glycoprotein.

As RC-101 was found to inhibit ZIKV infection at both the entry and replication stages (Fig. 2), we further investigated the mechanism underlying the inhibitory effect on the entry stage. As previously mentioned, RC has been reported to inhibit different types of enveloped viruses by binding to the negatively charged glycan chains on the surface of the glycoprotein, thus blocking virus entry (22–24). However, flaviviruses contain only one glycosylation motif on the E glycoprotein, but this the number is not absolutely conserved, as DENV has two glycosylation motifs, whereas some African linage ZIKV strains have no glycan chain on the surface (26–31, 37–39). As shown in Fig. 1, RC-101 exerted similar inhibitory effects on both the ZIKV Asian strain PRVABC 59 (one glycan) and the African strain MR766 (no glycan), suggesting that glycan might not be the target of RC-101. As RC-101 could block ZIKV infection at the entry stage (Fig. 2), we further investigated its effect on the E protein.

**FIG 5:**
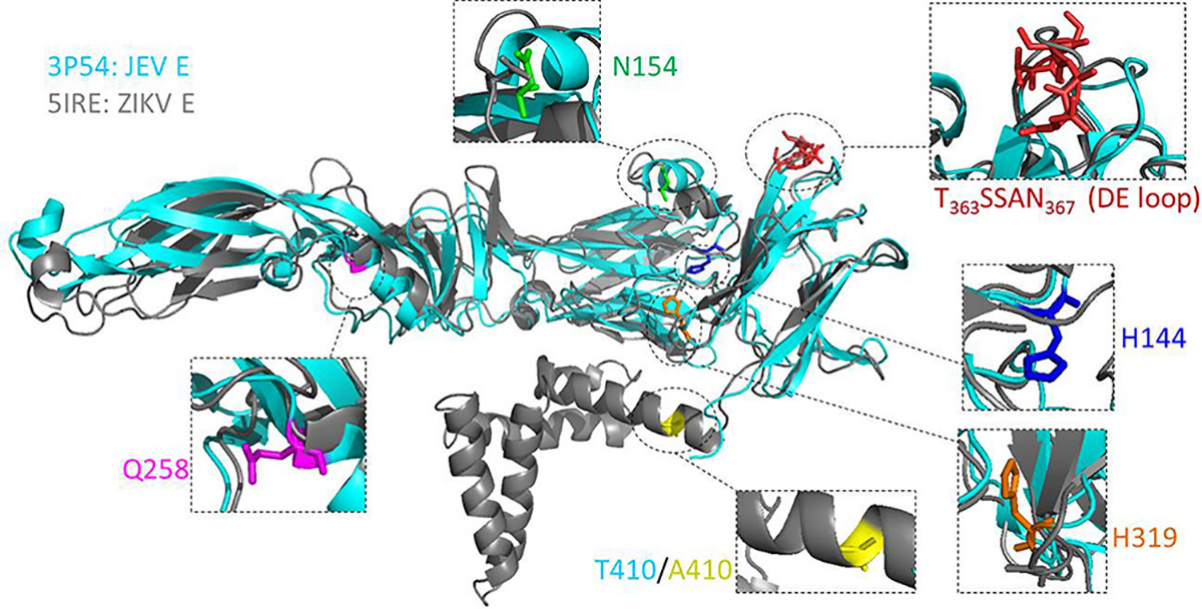
The potential viral target of RC-101 on flavivirus E protein. Shown is the side view of monomer prefusion Japanese encephalitis virus (JEV) E protein ectodomain conformation (cyan; PDB no. 3P54) in alignment with the full-length Zika virus (ZIKV) E protein (gray; PDB no. 5IRE). The potential targets tested in this study were enlarged and highlighted by color.

In our previously published work, we constructed a series of JEV variants with mutations in the receptor-binding motif or in amino acids critical for membrane fusion on the E protein (6). Considering the relative conservation of the sequence and structure of flavivirus E proteins, we used the constructed JEV variants to investigate the potential target of RC-101. Among the selected variants, the N154A and DE mutants (T_363_SSAN_367_ to A_363_AAAA_367_) impaired receptor binding by the virus, H144A and H319A abrogated the interaction between DI and DIII, and Q258A and T410A resulted in failure of the E protein to refold to form its postfusion conformation (6). Notably, these six tested sites were conserved between JEV and ZIKV (Fig. 5).

**FIG 6:**
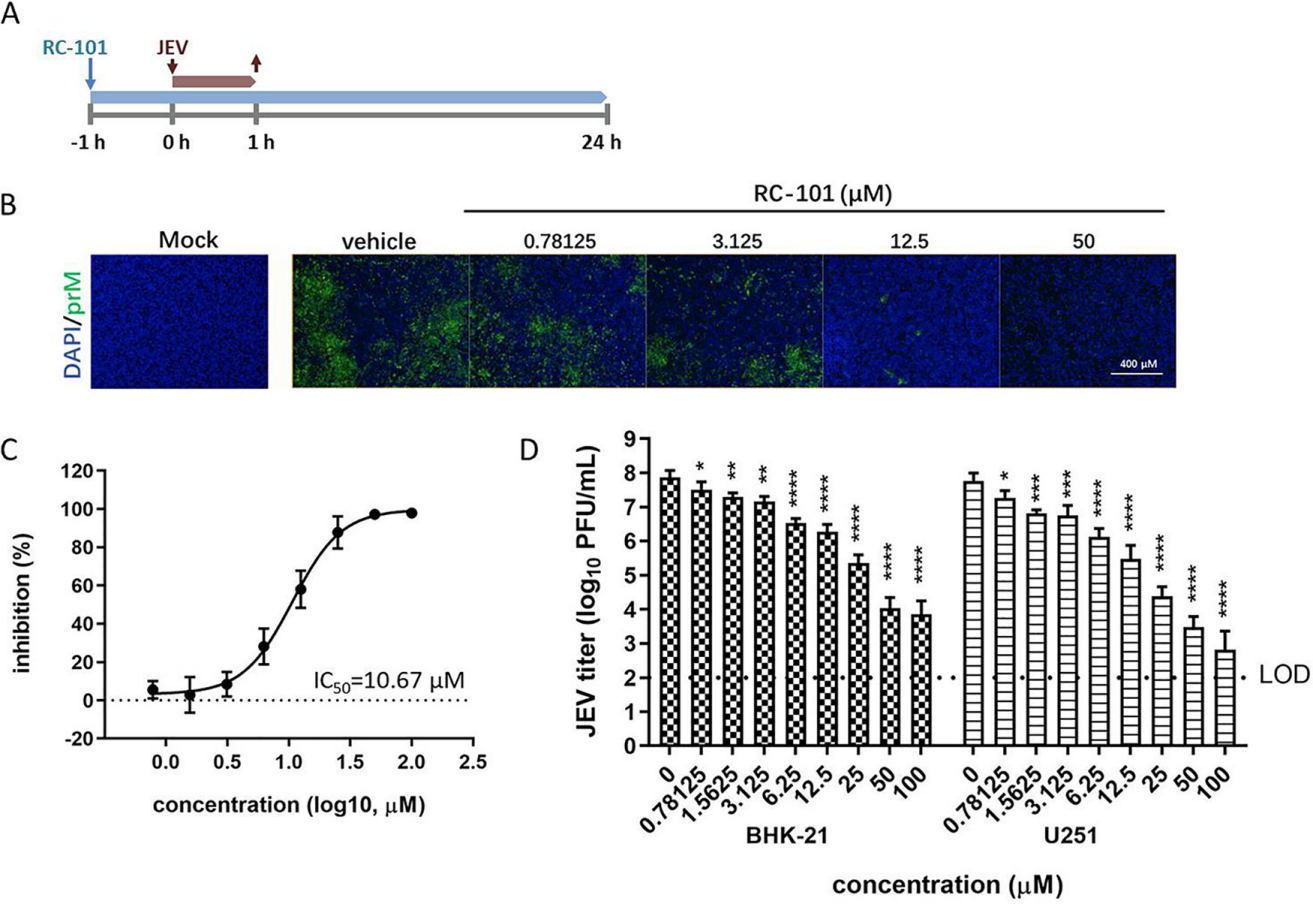
RC-101 inhibits JEV infection. (A) Timeline of the assay. Cells were incubated with RC-101 at the indicated concentrations from 1 h preinfection and then infected with JEV AT31 at an MOI of 0.1 for 1 h. (B) BHK-21 cells infected with JEV were analyzed for prM expression using the IFA assay 24 h postinfection. Cells were imaged using an Operetta high-content imaging system (PerkinElmer). (C) Dose-response curve based on the IFA results. The percentages of infected and DAPI-positive cells were calculated using the Harmony 3.5 software in the Operetta high-content imaging system. (D) The inhibition effects were validated in both BHK-21 and U251 cells using the plaque assay. Data are presented as the mean ± SD from six independent experiments. LOD, limit of detection. *, *P* < 0.05; **, *P* < 0.01; ***, *P* < 0.001; ****, *P* < 0.0001.

First, the antiviral effect of RC-101 against JEV was investigated. As shown in Fig. 6A to C, RC-101 dose-dependently inhibited JEV infection in BHK-21 cells, with an IC_50_ of 10.67 μM. Furthermore, the viral titer reduction assay confirmed that RC-101 robustly inhibited JEV infection in both BHK-21 and U251 cells (Fig. 6D).

**FIG 7:**
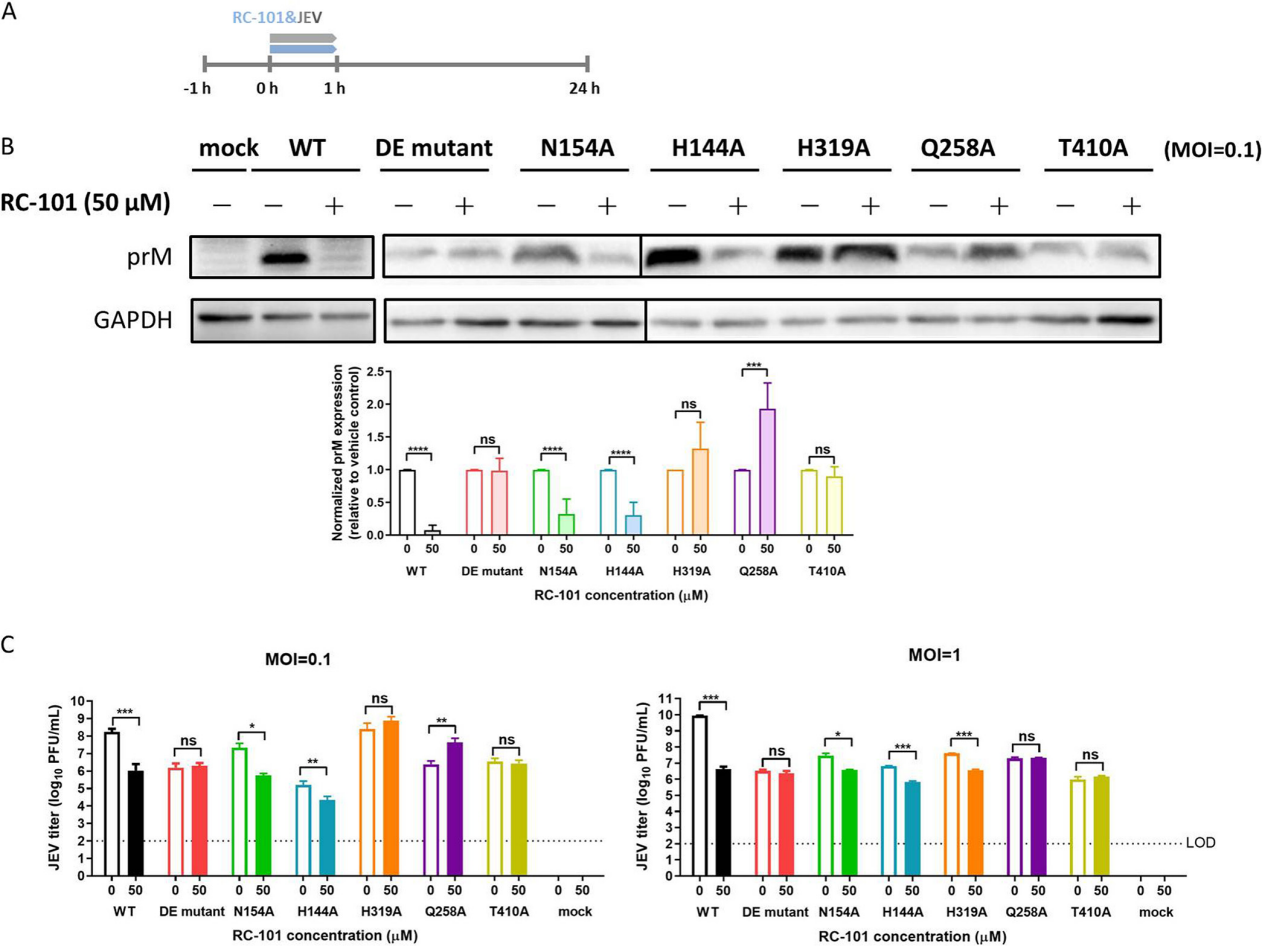
Sensitivity/resistance of the mutant viruses to RC-101. (A) Timeline of the assay. (B, top) JEV-infected BHK-21 cell lysates were analyzed by Western blotting at 24 h postinfection, and rabbit prM antiserum and the anti-GAPDH mouse monoclonal antibody were used as the primary antibodies (MOI of 0.1). (B, bottom) Quantification results of Western blotting are presented as the mean ± SD from 4 to 5 independent experiments. (C) The viral titers were tested by plaque assay using BHK-21 cells. Data are represented as the mean ± SD from 4 to 6 independent experiments. LOD, limit of detection. ns, not significant; *, *P* < 0.05; **, *P* < 0.01; ***, *P* < 0.001; ****, *P* < 0.0001.

The investigation was conducted using coadministration (Fig. 7A). As shown in Fig. 6B and C, RC-101 at 50 μM, corresponding to the approximate IC_98_ against ZIKV (Fig. 1), robustly inhibited JEV infection, which made the prM band hardly detectable, and the viral titers decreased by approximately 3 log units. Similarly, RC-101 inhibited infections by viruses harboring N154A and H144A, suggesting that neither N154 nor H144 is the target of RC-101. Of note, the outcome indicating that abolishing the glycosylation motif (N154A) resulted in retained sensitivity to RC-101 was in line with the notion that differences in the number of glycan chains in different strains have little effect on RC-101 inhibition (Fig. 1). This further confirmed that RC-101 has a unique antiflavivirus mechanism, which is unlike the effects on other enveloped viruses. Notably, as shown in Fig. 7B and C, the Q258A mutant likely had increased sensitivity to RC-101, whereas H319A resulted in resistance to RC-101 at the protein level and in the low-multiplication of infection (MOI) assay. Among the six tested mutants, the DE mutant and T410A showed robust resistance to RC-101 in all assays, indicating that these two mutants do confer resistance and might serve as the viral glycoprotein target(s) of RC-101. As T410 is located in the stem region of the E protein, buried by the compacted E dimer and hardly accessible in the prefusion conformation, the DE mutant was selected for further investigation of the binding affinity to RC-101.

### DE loop mutant decreases binding affinity to RC-101.

**FIG 8:**
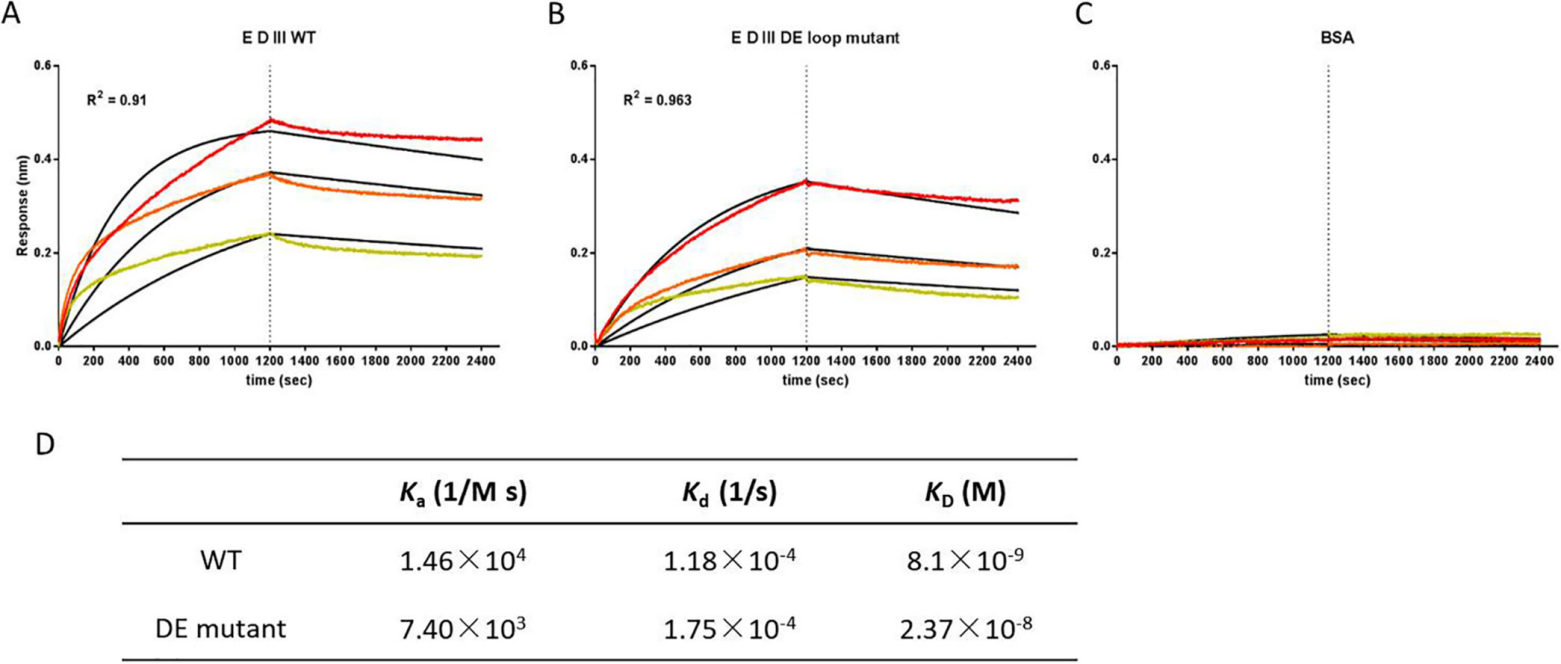
A DE loop mutation decreases the binding affinity of RC-101 to E protein domain III (DIII). WT DIII (A), DE loop mutant DIII (B), and bovine serum albumin (BSA) (C) were immobilized onto biosensors. The binding of RC-101 was assessed at 200 nM (red), 100 nM (orange), and 50 nM (yellow), and the global fit curves are shown as black lines. The vertical dashed lines indicate the transition between association and dissociation phases. (D) Binding affinities of WT and DE loop mutant DIII to RC-101.

To test the possibility that the DE loop is the target of RC-101, and to test whether the DE mutant would disrupt the binding of RC-101 to DIII, the binding affinities of the wild-type (WT) and the DE mutant DIII to RC-101 were examined by biolayer interferometry. The interactions between DIII and RC-101 were calculated using a 1:1 binding model at three different concentrations (Fig. 8). The results showed that RC-101 bound to WT DIII with a kinetic association (*K_a_*) of 1.46 × 10^4^ M^−1^ s^−1^, kinetic dissociation (*K_d_*) of 1.18 × 10^−4^ s^−1^, and an equilibrium dissociation constant (*K_D_*) of 8.10 × 10^−9^ M, indicating that RC-101 has high affinity for DIII. The binding affinity of RC-101 to the DE mutant was decreased by 1 order of magnitude, to a *K_D_* of 2.37 × 10^−8^ M, which suggested that the DE loop might be the binding site of RC-101 and that the DE mutant would disrupt this interaction.

## DISCUSSION

**FIG 9:**
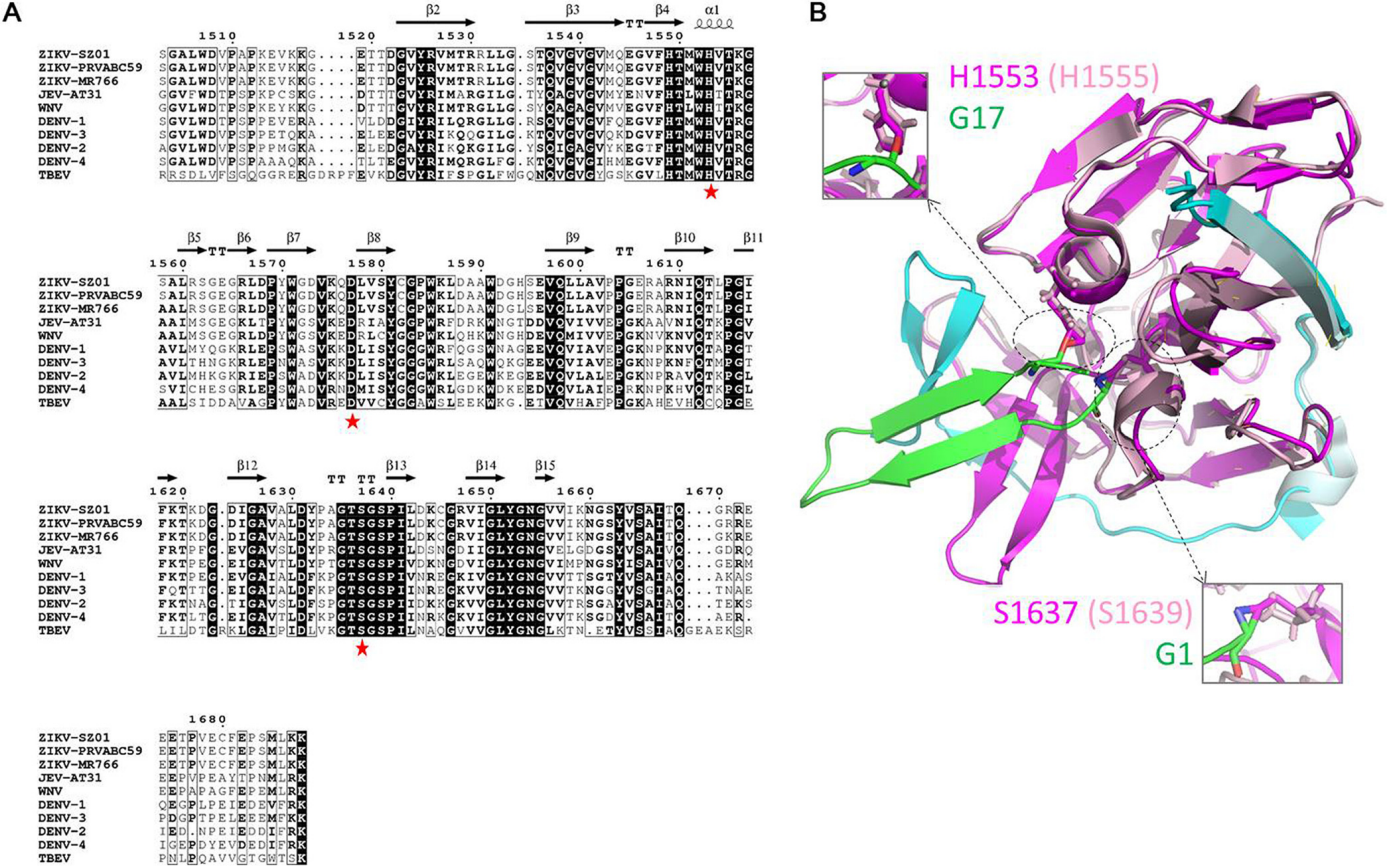
Docking of the NS2B-NS3/RC-2 complex. (A) Sequence alignment of the flavivirus NS3 N-terminal domain (positions 1503 to 1688). Secondary structure elements were graphically represented by ESPript (55) (http://espript.ibcp.fr). The secondary structure observed with Zika virus (ZIKV) NS2B-NS3 protease (PDB no. 5GXJ) is indicated above the sequence. The catalytic triad residues are indicated by red stars. The relevant GenBank sequence accession numbers are as follows: ZIKV strain SZ01, KU963796; ZIKV strain PRVABC 59, KU501215; ZIKV strain MR766, MK105975.1; Japanese encephalitis virus (JEV) strain AT31, AB196923.1; West Nile virus (WNV), NC_001563.2; dengue virus 1 (DENV-1), AY145122.1; DENV-2, NC_001474.2; DENV-3, MN227700.1; DENV-4, KY924607.1; and tick-borne encephalitis virus, MT311860.1. (B) Ribbon diagram of the NS2B-NS3/RC-2 complex. The crystal structure of RC-2 (PDB no. 2ZLI) and ZIKV NS3 (PDB no. 5ZMS) was used to build the complex using the ZDOCK 3.0.2 program. The crystal structure of JEV NS3 (PDB no. 4R8T) was aligned with that of ZIKV NS3. ZIKV NS2B, ZIKV NS3, JEV NS2B, JEV NS3, and RC-2 are colored cyan, magenta, pale cyan, light pink, and green, respectively. The supposed interacting residues between NS3 and RC-2 are shown as sticks.

Although RC has been reported to have inhibitory effects against different kinds of viruses with various antiviral mechanisms, few studies have investigated its effect on flaviviruses. In this study, we evaluated the antiviral effects of RC-101 against flaviviruses and elucidated the mechanism of action. As the analogue RC-1 has been reported to inhibit DENV NS2B-NS3 protease and viral replication, we first tested whether RC-101 could extend its antiviral spectrum to other flaviviruses. As a result, RC-101 was found to inhibit infections by different strains of ZIKV, as well as JEV. Furthermore, results suggest that the NS2B-NS3 protease might serve as one of the viral targets since RC-101 could block the serine protease activity of NS2B-NS3. The NS3 proteolytic domain forms a substrate-binding pocket with a catalytic triad, conserved in flaviviruses, of His-Asp-Ser (Fig. 9A). In an attempt to dock the analogue RC-2 (PDB no. 2LZI [GICRCICGRRICRCICGR]) (40) with ZIKV NS3 (PDB no. 5ZMS) (41), we found that glycine in RC-2 might interact with histidine (H1553) and serine (S1673) in the catalytic triad, and both of these residues are structurally conserved between ZIKV and JEV (Fig. 9B). RC-101 might thus inhibit NS2B-NS3 protease activity by competitively blocking the catalytic motif and thus preventing substrate binding. Meanwhile, as a cationic peptide, RC-101 might directly interact with the negatively charged NS2B and thus prevent the binding of NS2B and NS3 (20, 42).

As mentioned previously herein, RC has been extensively reported to inhibit enveloped viruses by targeting the negative glycan shield on the surface of the virus, thus blocking the initial entry of the virus into host cells (22–24). As the only glycan chain in the E protein of the ZIKV PRVABC 59 strain and JEV, the glycan linked to the N_154_YS glycosylation motif has been reported to interact with DC-SIGN, which is a candidate flavivirus receptor (43). Intriguingly, the N154A mutation had no impact on the sensitivity or resistance of JEV to RC-101. A possible explanation for this phenomenon is that RC-101 could easily bind with the dense glycan shield of gp120 and hemagglutinin (HA) of HIV and IAV, but in case of the flavivirus, RC-101 might pass through the unique glycan and interact with the E protein directly. The DE loop, which is the relatively higher tip of the E protein (Fig. 5), might serve as the viral target of RC-101. Although peptides derived from the DE loop were previously found to prevent JEV infection by interfering with virus attachment to BHK-21 cells (44), the DE loop is not the only or major receptor binding motif for JEV entry into different types of cells (6). Further studies should focus on whether RC-101 could inhibit flavivirus infection of different kinds of cells and whether the DE mutant confers resistance to RC-101 in other hosts and tissues.

Currently, there are no effective drugs approved for the treatment of flavivirus infection. Fortunately, several peptide inhibitors, derived from or targeting the E protein, have been used to successfully block flavivirus infection *in vitro* and *in vivo* (7, 9, 12, 45). As the flavivirus E protein has a highly conserved sequence and conformation, peptide inhibitors could be used for the treatment of emerging flavivirus infections or severe cases. In addition, peptide inhibitors have many advantages, such as high biocompatibility, a low frequency of selecting resistant mutants, the ability to synergize with conventional drugs, and activity toward multidrug-resistant virus strains (46). The cyclic peptide RC-101, with a unique structure that provides long-lasting protection against viral infection (47, 48), is a potential candidate for the development of a successful drug to treat flaviviruses and other infectious diseases.

## MATERIALS AND METHODS

### Cells, viruses, and RC-101.

Vero, BHK-21, and U251 cells were maintained in Dulbecco’s modified Eagle’s medium and minimum essential medium containing 10% fetal bovine serum (FBS), respectively. The ZIKV PRVABC 59 strains were kindly provided by Jean K Lim (Icahn School of Medicine at Mount Sinai, New York, NY) (GenBank accession no. KX377337.1) and Tong Cheng (School of Life Sciences, Xiamen University, China) (GenBank accession no. KU501215), while the MR-776 strain (GenBank accession no. MK105975.1) was obtained from The Microorganisms and Viruses Culture Collection Center, Wuhan Institute of Virology, Chinese Academy of Sciences. The genome sequence of ZIKV strain SZ-WIV001 (GenBank accession no. KU963796) was used as the template for the construction of the ZIKV replicon (49). JEV AT31 was generated using the infectious clones of pMWJEAT AT31 (kindly provided by T. Wakita, Tokyo Metropolitan Institute for Neuroscience) as previously described (50). The JEV variants, including the DE mutant N154A, H144A, H319A, Q258A, and T410A variants, were constructed and preserved at −80°C in our laboratory (6).

RC-101 was synthesized by solid-phase synthesis and purified by reversed-phase high-performance liquid chromatography (HPLC) to homogeneity (98% purity) (21). The effect of RC-101 on cell viability was evaluated using a cell counting kit (CCK-8) (Beyotime, Shanghai, China).

### Antiviral effects of RC-101.

Cells in 96-well plates were infected with ZIKV PRVABC 59, ZIKV MR-766, and JEV AT31 at the indicated MOI in the presence of RC-101 at different concentrations for 48, 72, and 24 h, respectively. The antiviral effects were evaluated by IFA assay and plaque assay.

### Primary antibodies.

Anti-ZIKV NS3 was a gift from Andres Merits, University of Tartu, Estonia, while the anti-GAPDH (anti-glycyeraldehyde-3-phosphate dehydrogenase) mouse monoclonal antibody was purchased from ABclonal (AC033, Wuhan, China). The anti-JEV prM polyclonal antibody was prepared by expressing full-length prM in Escherichia coli BL21 using a pET30a expression vector; purified protein was injected into rabbits to obtain the antiserum (6).

### IFA assay.

Cells were fixed with 4% paraformaldehyde, permeabilized using phosphate-buffered saline (PBS) containing 0.2% Triton X-100 for 15 min, and blocked with 5% fetal bovine serum (FBS; Gibco), followed by treatment with the primary antibody: anti-ZIKV NS3 or anti-JEV prM. After six rinses with PBS, the cells were stained with the secondary antibody, DyLight 488-labeled anti-rabbit IgG (KPL, Gaithersburg, MD, USA). Nuclei were then stained with DAPI (4′,6-diamidino-2-phenylindole) according to the manufacturer’s instructions (Sigma-Aldrich, USA). Nine fields per well were imaged using an Operetta high-content imaging system (PerkinElmer), and the percentages of infected and DAPI-positive cells were calculated using the associated Harmony 3.5 software.

### Western blotting.

JEV-infected BHK-21 cell lysates were analyzed at 23 h postinfection using rabbit prM antiserum, anti-JEV NS3 antibody (a gift from Bo Zhang, Wuhan Institute of Virology), and the anti-GAPDH mouse monoclonal antibody as primary antibodies.

### Plaque assay.

ZIKV and JEV were propagated in Vero cells and titrated in BHK-21 cells. The plaque assay was carried out by adding the serially diluted virus stock into semiconfluent monolayers of cells for 1 h. Then, the supernatant was discarded, and the cells were overlaid with medium containing 1% methylcellulose and incubated for the indicated time. The cells were then fixed with 4% formaldehyde and stained with 0.1% crystal violet for plaque visualization.

### Time-of-addition assay.

To determine which stage of the ZIKV life cycle was inhibited by RC-101, a time-of-addition experiment was performed as previously described (51). Vero cells were infected with ZIKV (MOI, 0.1) for 1 h (0 to 1 h). RC-101 (40 μM) was incubated with the cells for 1 h before infection (−1 to 0 h), coadministration infection (0 to 1 h), and for 47 or 71 h postinfection (1 to 48/72 h) (Fig. 2A). To exclude a possible direct inactivating effect of RC-101, ZIKV (MOI, 2.5) was incubated with RC-101 (40 μM) at 37°C for 1 h, and the mixtures were diluted 25-fold to infect Vero cells for 1 h. To confirm the inhibitory effect of RC-101 against ZIKV replication, BHK-21 cells were electroporated with the ZIKV replicon (SZ-WIV001; GenBank accession no. KU963796) and then incubated with RC-101. *Renilla* luciferase activity in the cell lysates was measured using the Rluc system (Promega, Madison, WI, USA) (52).

### Proteolytic activity of NS2B-NS3 protease.

To produce NS2B-GGGGSGGGG-NS3 protein, the ZIKV replicon was used as the template, and the NS2B fragments were amplified by PCR using a forward (5′-TTAAGAAGGAGATATACCATGGGCGTGGACATGTACATTGAAAGAG-3′) and reverse (5′-CACCACT*TCCACCTCCACCCGATCCACCTCCACC*GATCTCTCTCATGGGGGGACC-3′) primer pair, and NS3 was also amplified using a forward (5′-GAGATC*GGTGGAGGTGGATCGGGTGGAGGTGGA*AGTGGTGCTCTATGGGATGTGC-3′) and reverse (5′-CTCAGTGGTGGTGGTGGTGGTGCTCGAGCTTCTTCAGCATCGAAGGCTCGAAG-3′) primer pair. The underlined letters represent restriction endonuclease sites, and the italic letters represent the GGGGSGGGG linker sequence (20). The PCR products were cloned into pET28a using infusion PCR (Novagen, Darmstadt, Germany). The recombinant vector was transformed into E. coli BL21(DE3), and the cell lysates were loaded onto a nickel column. The protein was eluted with a gradient concentration of imidazole buffer (50 mM Tris-HCl, 30 mM NaCl, 50 to 500 mM imidazole [pH 7.0]) (35).

The proteolytic activity of NS2B-NS3pro was measured using a fluorescence resonance energy transfer-based assay with a fluorogenic peptide substrate (Boc-Gly-Arg-Arg-AMC; no. I-1565, Bachem) as the substrate. The relative fluorescence units were measured using an EnSpire multimode plate reader with the emission at 440 nm upon excitation at 350 nm. The kinetic parameter of NS2B-NS3pro was obtained using substrate from 2.5 to 20 μM in the fluorescent assay after a 30-min incubation at 37°C (20, 53). The *K_m_* was calculated from a combination of enzyme kinetics and velocity as a function of the substrate model using GraphPad Prism 8.0. The inhibitory effects of RC-101 against protease activities were assessed at 37°C for 30 min, with mixtures of 100 μl consisting of 12 μM fluorogenic peptide substrate, 1.25 μM NS2B-NS3pro, and RC-101 at concentrations ranging from 0 to 100 μM, buffered at pH 8.5 with 200 mM Tris-HCl. The IC_50_ value of RC-101 was evaluated using the nonlinear regression model in GraphPad Prism 8.0.

### Expression of WT and DE mutant DIII.

The WT DIII expression vector was constructed using pET-22b(+) and preserved in our laboratory (7). The DE mutant was constructed using the East Mutagenesis System kit (TransGen Biotech, China) with the following primer pair: forward, 5′-CAGTGAACCCCTTCGTCGCGGCGGCGGCGGCGGCGTCAAAGGTGC-3′; reverse, 5′-CGCCGCCGCCGCCGCCGCGACGAAGGGGTTCACTGTCACCAGCCG-3′ (6). WT DIII was expressed using E. coli BL21(DE3); the supernatant of the bacterial pellets was loaded onto a nickel column, and the bound protein was eluted with a gradient concentration of imidazole buffer. DE mutant DIII, expressed as inclusion bodies, was solubilized in 8 M urea (50 mM Tris-HCl, 100 mM NaCl, 1 mM dithiothreitol [DTT], 0.1% SDS, 8 M urea, pH 7.4). Refolding was carried out by titration dialysis at 4°C against refolding buffer (50 mM tris-HCl, 100 mM NaCl, 0.1% SDS, 1 mM l(+)-arginine, 1 mM glutathione, 5% glycerol [pH 7.4]) until the concentration of urea was <2 M. Then, the supernatant was passed through a nickel column as described previously herein.

### Binding affinity assay.

Real-time binding assays between RC-101 and WT or the DE mutant DIII were performed using biolayer interferometry on an Octet QK system (Fortebio, USA) according to previously reported methods (7). Binding kinetics were calculated using the Octet QK software package, which fit the observation to a 1:1 model to calculate the association and dissociation rate constants. Binding affinities were calculated as the *K_d_* rate constant divided by the *K_a_* rate constant.

### Docking of the NS2B-NS3/RC-2 complex.

The crystal structures of RC-2 (PDB no. 2ZLI) and ZIKV NS3 (PDB no. 5ZMS) were used to build the complex using the ZDOCK 3.0.2 program (http://zdock.umassmed.edu) (54). The resulting model was represented by PyMOL (Fig. 9).
